# A Multimodality Myocardial Perfusion Phantom: Initial Quantitative Imaging Results

**DOI:** 10.3390/bioengineering9090436

**Published:** 2022-09-04

**Authors:** Marije E. Kamphuis, Henny Kuipers, H. Remco Liefers, Jan van Es, Frank F. J. Simonis, Marcel J. W. Greuter, Cornelis H. Slump, Riemer H. J. A. Slart

**Affiliations:** 1 Multimodality Medical Imaging Group, Faculty of Science and Technology, University of Twente, 7500 AE Enschede, The Netherlands; 2 Robotics and Mechatronics Group, Faculty of Electrical Engineering Mathematics and Computer Science, University of Twente, 7500 AE Enschede, The Netherlands; 3 Department of Radiology, Medisch Spectrum Twente, 7512 KZ Enschede, The Netherlands; 4 Technical Medical Simulation Centre, Faculty of Science and Technology, University of Twente, 7500 AE Enschede, The Netherlands; 5 Magnetic Detection and Imaging Group, Faculty of Science and Technology, University of Twente, 7500 AE Enschede, The Netherlands; 6 Department of Radiology, University Medical Center Groningen, University of Groningen, 9700 RB Groningen, The Netherlands; 7 Department of Nuclear Medicine and Molecular Imaging, University Medical Center Groningen, University of Groningen, 9700 RB Groningen, The Netherlands; 8 Biomedical Photonic Imaging Group, Faculty of Science and Technology, University of Twente, 7500 AE Enschede, The Netherlands

**Keywords:** phantom, myocardial, perfusion, blood flow, quantitative imaging, multimodality, PET, CT, MRI

## Abstract

This proof-of-concept study explores the multimodal application of a dedicated cardiac flow phantom for ground truth contrast measurements in dynamic myocardial perfusion imaging with CT, PET/CT, and MRI. A 3D-printed cardiac flow phantom and flow circuit mimics the shape of the left ventricular cavity (LVC) and three myocardial regions. The regions are filled with tissue-mimicking materials and the flow circuit regulates and measures contrast flow through LVC and myocardial regions. Normal tissue perfusion and perfusion deficits were simulated. Phantom measurements in PET/CT, CT, and MRI were evaluated with clinically used hardware and software. The reference arterial input flow was 4.0 L/min and myocardial flow 80 mL/min, corresponding to myocardial blood flow (MBF) of 1.6 mL/g/min. The phantom demonstrated successful completion of all processes involved in quantitative, multimodal myocardial perfusion imaging (MPI) applications. Contrast kinetics in time intensity curves were in line with expectations for a mimicked perfusion deficit (38 s vs. 32 s in normal tissue). Derived MBF in PET/CT and CT led to under- and overestimation of reference flow of 0.9 mL/g/min and 4.5 mL/g/min, respectively. Simulated perfusion deficit (0.8 mL/g/min) in CT resulted in MBF of 2.8 mL/g/min. We successfully performed initial, quantitative perfusion measurements with a dedicated phantom setup utilizing clinical hardware and software. These results showcase the multimodal phantom’s potential.

## 1. Introduction

Myocardial perfusion imaging (MPI) at rest and under stress is a functional imaging method widely used to assess ischemia and confirm the diagnosis of coronary artery disease [1]. This imaging approach is pre-eminently performed and validated using single-photon emission computed tomography (SPECT) [2] and to an increasing extent by positron emission tomography (PET) [3]. Dynamic image acquisition of contrast media kinetics, including radiolabeled pharmaceuticals, can improve standard, relative perfusion evaluation. Numerous mathematical blood flow models have been developed to estimate local perfusion values from the imaged contrast properties over time [4,5,6]. Other quantitative measures indicating perfusion deficit, including a prolonged time to peak, can be derived from subsequent time intensity or time activity curves (TICs/TACs) as well. At the moment, the added value of (absolute) perfusion quantification in addition to standard relative assessment has been carefully investigated merely using PET imaging [7]. It can be concluded from these studies that estimation of myocardial blood flow (MBF), in particular myocardial flow reserve (MFR), can lead to improved diagnostic accuracy in certain patient groups and result in better and standardized evaluation capabilities [8]. However, caution is warranted when it comes to embedding absolute perfusion measures into clinical routine. Validation and harmonization of measurements is essential before these it can optimize revascularization decision making [9]. Next to PET, absolute MPI is also emerging with other imaging modalities, such as SPECT [1,10], computed tomography (CT) [11], magnetic resonance imaging (MRI) [12,13], and ultrasound [14,15]. Due to this diversity in the clinical setting, it is warranted to investigate perfusion measurement (in)accuracy in a controlled environment preceding a validated standard for quantitative, multimodal MPI.

It is relevant to study clinical applications of quantitative MPI in a simplified and controlled environment. In previous work [16], we presented a dedicated left ventricular flow phantom in which we introduced the use of sorbents to mimic radiolabeled contrast media uptake and retention in the simulated myocardium. We performed several ground truth tracer kinetic phantom measurements in dynamic SPECT and obtained promising, patient-realistic TACs (i.e., comprising arterial input functions (AIFs) and tissue response functions (TRFs)) of normal perfusion levels and regional or global perfusion deficits. One of the next steps in phantom development is to expand the current application domain to multiple imaging modalities, including associated contrast kinetics. Hence, the two main goals of this explorative study are: (1) to gain insight into the overall functionality of the phantom in quantitative MPI applications with PET/CT, CT, and MRI and (2) to identify possible improvements in phantom design.

## 2. Materials and Methods

### 2.1. Phantom Setup

The standard phantom setup comprises 3 elements:

1. A previously adapted myocardial perfusion phantom (cylindrical cardiac insert);
2. A commercial anthropomorphic thorax phantom;
3. An in-house built flow circuit.

**Figure 1:**
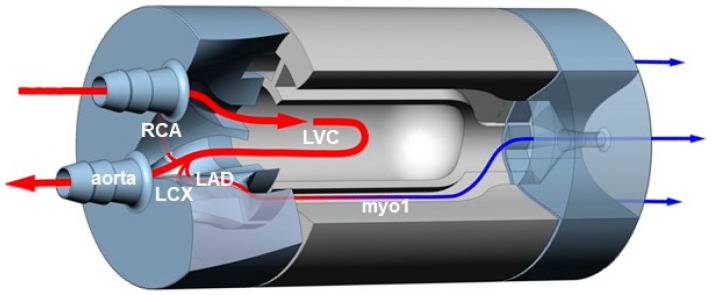
Tailored cross-section of the myocardial perfusion phantom design, consisting of three cylindrical parts fastened together with nylon screws. The middle phantom part comprises the simulated left ventricular cavity (LVC) and three surrounding myocardial regions (myo1–3). The two outer parts contain the in- and outlet connections to the flow circuit and the internal branches from the simulated aorta to myo1–3 (only myo1 is visible in this view). The branches correspond to the main coronary arteries, i.e., the left anterior descending coronary artery (LAD), right coronary artery (RCA) and left circumflex coronary artery (LCX). The arrows indicate flow direction and magnitude, and its color differentiates between arterial input (red) and venous output (blue).

Detailed information on the phantom setup can be found in previous work by Kamphuis et al. [16]. Briefly, the stationary, 3D-printed myocardial perfusion phantom represents the basic shape of a left ventricle. As visualized in Figure 1, the phantom assembly consists of three individual parts. The phantom has one inlet to the mimicked left ventricular cavity (LVC) and one main outlet representing the aorta. The aorta branches to three mimicked coronary arteries, namely the left anterior descending coronary artery (LAD), the right coronary artery (RCA), and left circumflex coronary artery (LCA). These branches connect to three identical, surrounding myocardial volumes. Each volume exists of an individual outlet. The tissue mimicking material used to fill these volumes is linked to the intended contrast/tracer kinetics to be simulated.

**Figure 2:**
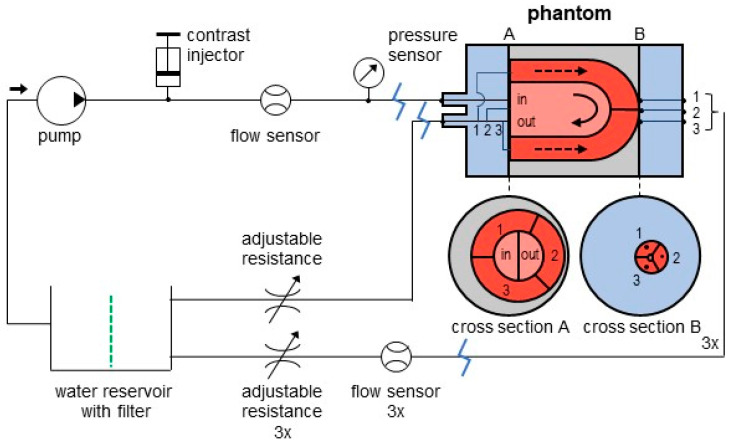
Flow circuit design of the standard phantom setup. The 3D printed phantom cylinder exists of one inlet to the simulated left ventricular cavity. Its main outlet (mimicking the aorta) branches to three identical surrounding myocardial regions (1–3). Each region has an individual outlet. All flow circuit components are connected by silicon tubing. Modality-specific adjustments incorporate a custom-built filter in the water reservoir to adsorb recirculating radiotracer during SPECT and PET measurements (dashed green line) and additional tubing of 8 m was built in at the in blue marked positions for MRI measurements. The arrow in the left top indicates flow direction.

The closed-loop flow circuit connects to all phantom inlets and outlets (see Figure 2). Using this flow circuit, we can generate, regulate, and measure water flow (with injected contrast agent or radiotracer) through the LVC and individual myocardial regions. Tuning of the resistances/taps in the flow circuit enables simulation of normal tissue perfusion and regional or global perfusion deficits. Standard flow settings were 4.0 L/min as cardiac output and 80 mL/min within each myocardial region. The latter corresponds to a myocardial blood flow of 1.6 mL/g/min (normalized by tissue weight, with an assumed tissue density of 1.0 g/mL and a volume of 50 mL per myocardial region).

**Figure 3:**
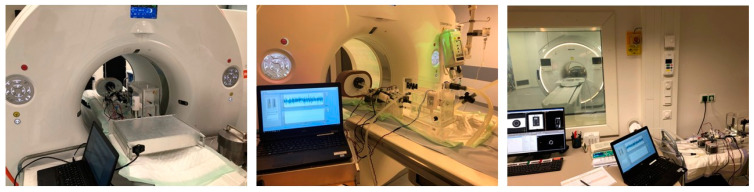
Example of the overall phantom measurement setup in PET/CT (**left**), CT (**middle**) and MRI (**right**).

In all phantom measurements, we aimed to image first pass, patient-realistic contrast kinetics through the mimicked LVC and myocardial regions over time. The same basic measurement setup, as illustrated in Figure 3, was used for all phantom measurements. The following modality specific adjustments were made regarding the flow circuit and the used tissue mimicking material.

### 2.2. Flow Circuit Adjustments

The described flow circuit was designed for use with [99mTc]pertechnetate and SPECT imaging [16]. In this setup, a closed loop configuration was preferred in order to avoid large amounts of radio-active wastewater. Use of a custom-built filter (adsorbing passing radiotracer) prevents recirculation of radiotracer and hence aims for the intended measurement of first pass tracer kinetics. No adjustments to the flow circuit seemed necessary for phantom measurements with PET/CT imaging as we assumed the existing recirculation filter applied in SPECT would be sufficient for PET/CT as well [17]. The filter comprised of 1 kg of aquarium filter material, which is a mixture of zeolite (~50%) and activated carbon (~50%) (Superfish Crystal Clear Media, AquaDistri, Klundert, The Netherlands). Previous experiments have shown adsorption of passing [99mTc]pertechnetate using activated carbon [16], and it was hypothesized that zeolite would adsorb passing [13N]ammonia satisfactorily [17], provided that a sufficient amount of filter material was used. Small adjustments to the closed loop flow circuit were necessary for phantom measurements with CT, because no adsorption of an iodine-containing contrast medium was attained in the lab using current filter design. These adjustments included: (1) enlargement of the water reservoir (5 L in total) such that recirculation of contrast falls outside the 30 s scanning time and (2) replacement of the iodine containing water with fresh tap water in between phantom measurements (i.e., strong dilution) to prevent increasing contrast concentrations being imaged over time. For MR measurements, the setup was placed outside the Faraday cage. Only the phantom itself was positioned on the scanning table by means of 5 silicone tubes of 8 m (see Figure 2 and Figure 3). The phantom cylinder was placed in a water container. For the same reason as with the CT measurements, the contrast containing water was replaced by fresh tap water in between measurements.

### 2.3. Tissue Mimicking Adjustments

Ideally, a dedicated myocardial perfusion phantom can mimic a standard course of a specific (radiolabeled) contrast medium. Previous phantom measurements with SPECT have shown that the concept of using sorbents as tissue mimicking material offers novel possibilities to mimic contrast specific kinetics [16]. We demonstrated with SPECT-MPI that a certain amount of activated carbon, placed in the myocardial tissue volumes of the phantom, can provide standardized myocardial uptake of [99mTc]pertechnetate in accordance with normal perfusion levels. In principle the same can be accomplished with other contrast media and for other imaging modalities. For phantom measurements with PET/CT, we poured different amounts of zeolite, supplemented with activated carbon or silica granulates, into three myocardial tissue volumes. This proof-of-concept study was performed to explore whether trapping of [13N]ammonia can be attained and modified accordingly. For CT measurements, we used sponge as tissue mimicking material [18,19,20,21]. Iodine based contrast media hardly leaves the microvasculature and therefore no trapping has to be simulated. For MRI measurements, there were no specific requirements set for tissue-mimicking properties yet, so we used a mixture of activated carbon and silica granulate for this feasibility experiment.

### 2.4. Phantom Measurements

Initial measurements were aimed at describing to what extent the current phantom setup can be used for multimodal, quantitative MPI evaluation. In this, we went through all steps along the dynamic MPI procedure: from patient/phantom preparation, image acquisition, and reconstruction to perfusion analysis and reporting on the feasibility per modality. A total of nine phantom measurements were performed on three different scanners (3 in PET/CT, 3 in CT, and 3 in MRI) following clinical MPI protocols where possible. An overview of the study variables is listed in Table 1. At the start, the phantom setup was built up on the scanning table in a standard manner. Then, the flow circuit was filled with tap water and vented, connected to the clinical contrast injector, and set to standard flow settings (4.0 L/min through LVC and 3 × 80 mL/min through the myocardial regions). Hereafter the clinical scanning procedure could be started.

### 2.5. Dynamic Myocardial Perfusion Imaging

#### 2.5.1. PET/CT Imaging

The phantom study was performed on a digital PET/CT scanner (Vision Biograph PET/CT 128-Multislice Scanner; Siemens Healthineers, Erlangen Germany). Myocardial perfusion was assessed three times with a dose of 400 MBq [13N]ammonia injection. CT-based transmission scans (120 kVp; 20–30 mA; pitch 1.5) were obtained prior to the first perfusion study to determine the field of view (FOV) and evaluate the presence of unwanted air bubbles, followed by venting efforts until this was resolved satisfactorily. It should be noted that for PET/CT measurements, an unforeseen resistance in one phantom region resulted in a set flow of 40, 80, and 80 mL/min in myocardial regions 1–3, respectively. Then, dynamic perfusion imaging data were obtained for 8.5 min and visualized in 25 frames (1 × 7.5 s, 11 × 5 s, 1 × 7.5 s, 1 × 10 s, 1 × 20 s, 6 × 30 s, 1 × 45 s, 1 × 60 s, and 1 × 120 s). A standard reconstruction (2D attenuation-weighted OSEM) was used with 3 iterations, 14 subsets, and 3D post-filtering with a 5 mm Gaussian filter kernel. Transverse data were reformatted to a 168 × 168 × 47 matrix with 2 mm pixels for each dynamic frame.

#### 2.5.2. CT Imaging

Dynamic CT perfusion scans were performed at a third-generation dual source CT scanner (SOMATOM Force, Siemens Healthineers, Forchheim, Germany). The FOV was determined based on frontal and lateral scout images and covered the anthropomorphic thorax phantom with myocardial perfusion phantom insert. Dynamic scans were started nine seconds prior to contrast bolus injection into the flow circuit. 60 mL of 350 mg/mL iomeprol solution (Iomeron350, Bracco, Milan, Italy) was injected at an injection rate of 6.0 mL/s. Contrast injection was followed by a 32 mL saline flush, also at 6.0 mL/s. Based on preliminary phantom measurements we slightly altered the standard contrast protocol (40% dilution with saline) to obtain patient realistic AIFs (i.e., a peak intensity of around 800 HU). Dynamic CT perfusion scans of 30 s were performed in shuttle mode during end-systole (mimicked by a simulated 60 bpm ECG trigger pulse), providing one full heart coverage scan with a z-range of 102 mm per 2 s. Other acquisition parameters included a tube voltage of 70 kVp, a tube current time product of 280 mAs, and a gantry rotation time of 0.6 s. Dynamic CT perfusion data were reconstructed with 3.0 mm slice thickness and 1.5 mm increment. Traditional filtered back projection was used with a B23f kernel.

#### 2.5.3. MR Imaging

Dynamic image acquisition was performed using a 1.5 T clinical MRI scanner (Ambition X, Philips, The Netherlands) whereby the same anterior and posterior receive coils were used as clinically used for patient scanning. In this, the predetermined FOV (210 mm × 252 mm) covered the water tank in which the phantom was positioned. Then, a clinical Balanced Turbo Field Echo sequence was performed. According to clinical protocol, 7.5 mmol of gadolinium (Dotarem, Guerbet, Roissy, Cedex, France) was dissolved in 15 mL saline and injected simultaneously with the start of the sequence at a flow rate of 3.0 mL/s, followed by a saline end-flush of 20 mL, injected at the same speed. Dynamic perfusion image series of 1 frame/s were acquired for a scan duration of 60 s in four manually selected image planes. These image planes included three short axis planes from heart base to apex and one of the 4-chamber planes. The image data has a voxel size of 2.02 mm by 2.23 mm (matrix of 104 × 113 voxels) and a slice thickness of 10 mm.

### 2.6. Perfusion Analysis

#### 2.6.1. PET/CT Analysis

All PET/CT images were processed with SyngoMBF VB14 (Siemens Healthineers, Forchheim, Germany). Dynamic datasets were automatically loaded, centered, and oriented, followed by manual corrections for adjustment of centering and reorientation of axial limits only when needed. The two-tissue compartment model developed by Hutchins et al. [22] was fitted to measure the TACs in order to calculate segmental MBF (presented in polar maps). In this, the arterial input function was derived by an automatically drawn region of interest at the base of the delineated LV. TRFs were extracted from standard delineated myocardial segments according to the American Heart Association’s 17-segment model [23]. In this, segments 7, 9, and 11 corresponded to the center of the three phantom tissue regions. For the second and third scan, measured background activity in the myocardial regions was subtracted using a standard tool in the clinical software. In this, the activity measured in the last frame of the previous study was subtracted from all frames in the following study. Multimodality TIC/TAC data was exported to Matlab2021 (MathWorks, Inc., Natick, MA, USA) for visualization purposes.

#### 2.6.2. CT Analysis 

CT images were processed with Syngo.via Enterprise Browser VB40 HF20 (Siemens Healthineers, Forchheim, Germany). The dynamic datasets were automatically loaded, centered, and oriented. As a next step, an intensity threshold was placed to differentiate arterial input flow data from surrounding tissue. Then two regions of interest (ROIs) were manually drawn in two image slices (comprising both shuttle modes) to derive an average AIF from the ROIs in the LVC. Next, three similar tissue volumes of interest (VOIs) were delineated in different image slices corresponding to the three myocardial regions, after which the software automatically contoured the entire VOIs. Based on the intensity profiles within the VOIs, i.e., the TRFs, the software automatically calculated local and regional MBF values using a maximum slope method. Such a method is derived from the Fick principle and calculates MBF by dividing the maximum gradient of the TRF by the peak of the AIF [24,25].

#### 2.6.3. MR Analysis

The acquired images were analyzed using Intellispace software (Philips Healthcare, Eindhoven, The Netherlands). The endocardial and epicardial contours were manually drawn in a single basal, mid ventricle, and apical slice. These were then automatically propagated to all dynamic phases. As a next step, a ROI was automatically drawn in the center of the endocardial contour to generate an AIF. Subsequently, a 6-tissue segment model was superimposed over the image slices in the short axis views to generate segmental TRFs. The orientation of this segment model was manually oriented to match with the myocardial regions in the phantom.

## 3. Results

**Figure 4:**
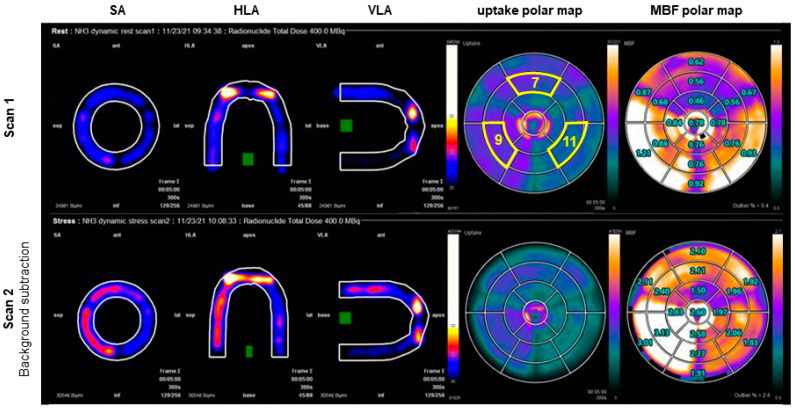
Two PET-CT myocardial perfusion imaging scans. The cross-sections along the sagittal axis (SA), horizontal longitudinal axis (HLA) and vertical longitudinal axis (VLA) display accumulated radiotracer activity. The green boxes indicate the regions of interest that were used to calculate the arterial input function. The polar plots show myocardial tracer uptake (left) and computed myocardial blood flow (MBF) (right). The three depicted heart segments (circled in yellow in the top uptake polar map) represent the center of the three phantom tissue regions (myo1–3) and were used for further tissue response function and MBF analysis.

**Figure 5:**
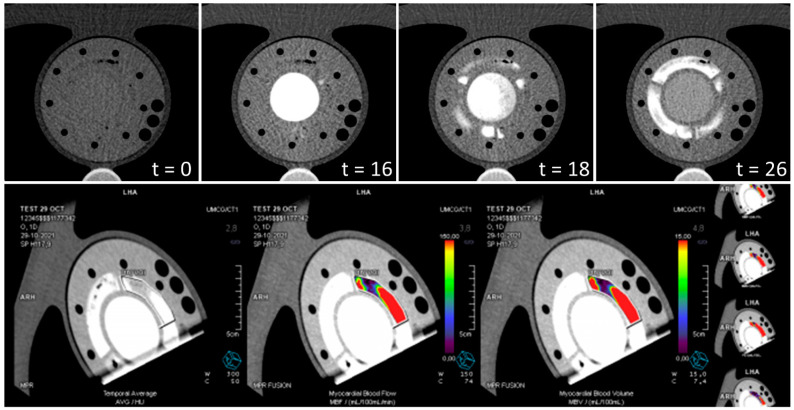
Example of CT myocardial perfusion image analysis. (**Top**) visualization of a time-lapse across a short axis plane of the phantom showing contrast arrival and distribution in the mimicked left ventricular cavity (center) and three surrounding myocardial regions (t = 0 s, 16 s, 18 s, 26 s). The rightmost tissue region simulates a perfusion deficit (flow of 40 mL/min instead of 80 mL/min). (**Bottom**) a typical software display of CT myocardial perfusion image analysis in consecutive image slices. The level of estimated myocardial blood flow within the manually delineated volume of interest is visualized by the added color and ranges from 0 (purple) to 150 mL/100 mL/min (red).

**Figure 6:**
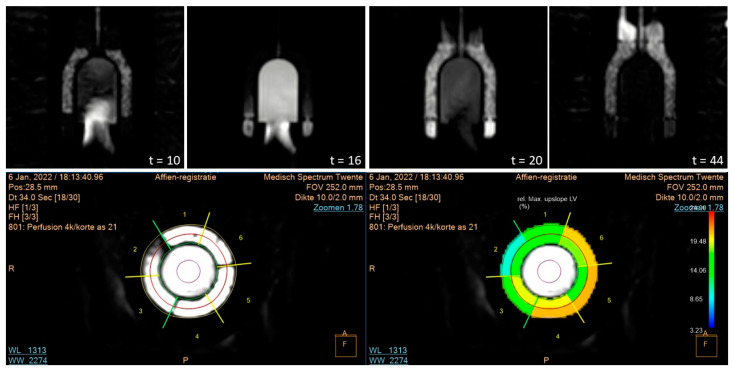
Example of MR myocardial perfusion image analysis. (**Top**) visualization of a time-lapse across a longitudinal heart axis plane showing contrast arrival and distribution in the mimicked left ventricular cavity (center) and three surrounding myocardial regions (time in seconds). (**Bottom**) clinical software display of segmental myocardial perfusion analysis. In this, segment 2 depicts a simulated perfusion deficit. The colors indicate the relative maximum upslope of the tissue region as a ratio of the maximum upslope in the left ventricular cavity, scaled from 3.23 to 24.90%.

Figure 4 illustrates clinical analysis of two dynamic PET-MPI data sets. The use of different tissue mimicking material compositions resulted in varying tracer uptake as shown in the uptake polar maps. The tissue region depicted by heart segment 9 showed the highest tracer uptake level, corresponding to myo2 in the phantom. Tracer uptake was almost absent in segment 11, which matched myo3. The second row in Figure 1 plots a repeated phantom measurement with background subtraction of previously accumulated radiotracer. Scan 1 and 2 showed a similar relative tracer uptake distribution across the mimicked myocardial tissue, though deviated from each other in terms of absolute computed MBF values. Figure 5 demonstrates myocardial perfusion image visualization and analysis in CT. The top row illustrates a phantom image time-lapse across the short axis showing contrast arrival and distribution within the LVC and myocardial regions. A perfusion deficit was simulated in the rightmost tissue region. Figure 5 displays typical MBF analysis of a VOI in consecutive image slices. An example dynamic phantom image series obtained with MRI is visualized in the top row of Figure 6. Underneath a typical (partial) software display is presented for subsequent perfusion analysis.

**Figure 7:**
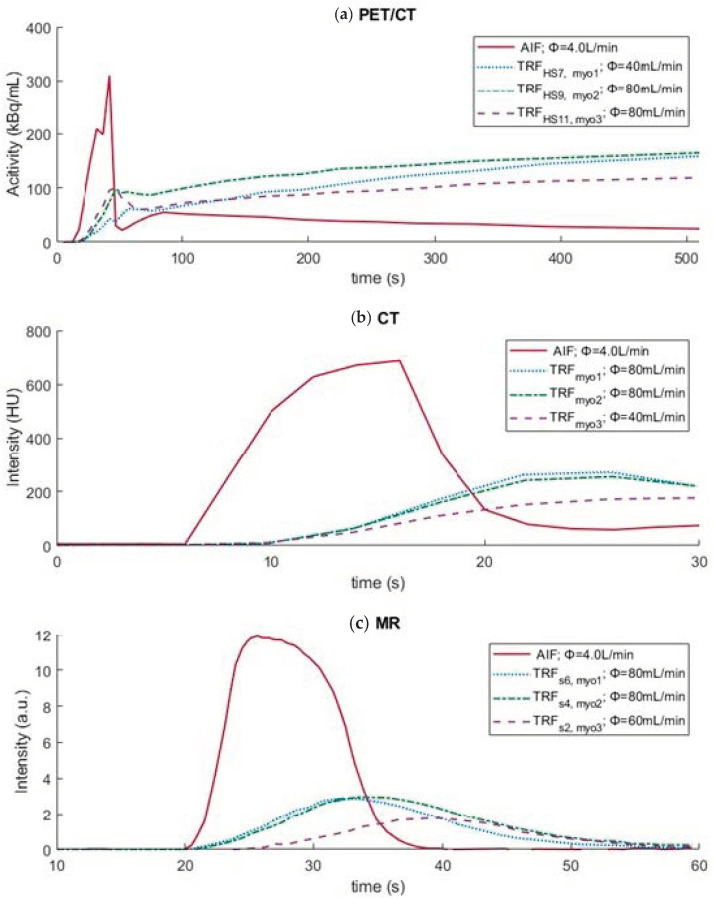
Example time activity/intensity curves for (**a**) PET/CT, (**b**) CT, and (**c**) MR phantom measurements displaying the arterial input function (AIF) and regional tissue response functions (TRFs). For PET/CT, these regions comprised heart segments (HS) 7, 9, and 11, which matched the center of the three phantom myocardial regions (myo1–3). For CT these regions comprised myo1–3 in its total (manually delineated), and for MR the segments (s) comprised parts of myo1–3. Note: the x-axes have different time scales.

Multimodal TACs/TICs are displayed in Figure 7, highlighting contrast media distribution over time within the mimicked LVC and myocardial tissue regions/segments. Table 2 provides an impression of software derived MBF values (from a single scan) compared to the set reference flow in the phantom setup.

## 4. Discussion

This explorative study had two main goals: first, to study overall functionality of our dedicated myocardial perfusion phantom for multimodal use and, second, to identify possible improvements in the phantom setup design. The phantom results obtained with PET/CT, CT, and MRI demonstrated successful completion of all the steps involved in these quantitative MPI applications: from dynamic image acquisition of contrast kinetics to subsequent perfusion analysis (see multimodal software displays, Supplemental Digital Content S1). We now further elaborate on the executed steps per imaging modality.

With the PET/CT phantom measurements, we wanted to verify whether it is possible to mimic myocardial uptake of [13N]ammonia using sorption technology, and if so, whether the subsequent accumulated detection of tracer activity in the phantom leads to recognition and mapping of patient realistic heart contours by clinical analysis software. These initial results demonstrated that zeolite indeed provides a certain amount of [13N]ammonia trapping, which appears to be controllable by using different tissue mimicking material compositions (Figure 4 and Figure 7). As a result, the phantom heart contours were well recognized by the software, as with previous SPECT measurements [16,26]. No activity can occur at the location of the partitions in the myocardial tissue volumes of the phantom. We have used these markings in the polar maps to facilitate standardized orientation and evaluation. Manual adjustments in phantom orientation could be easily applied in the software. As a result, heart segments 7, 9, and 11 could be selected for further TAC and MBF analysis. These segments occupied the center of the myocardial volumes in the phantom. As can be noticed in Figure 7, the TRF of heart segment 7 revealed a somewhat delayed uptake of radiotracer due to the lower flow setting. The stronger increase of this curve compared to the other TRFs can be explained by the larger amount of adsorption material present in this segment. Overall, the phantom TACs showed good resemblance with patient TACs [27]. Further research in finding the adequate tissue-mimicking material composition and standardized fabrication may further enhance tracer kinetic phantom measurement of [13N]ammonia up to the level of two-compartmental blood flow analysis. Furthermore, we minimized adjustments to the closed flow circuit and used the same recirculation filter as applied in previous measurements with SPECT. However, we can derive from the slightly increasing TACs in Figure 7 that using this setup a small amount of [13N]ammonia recirculated throughout the scan time at t > 50 s. An improved filter design should encounter for measurement and analysis of more controlled, first pass tracer kinetics. In line with this, we can also argue that current background subtraction method (provided by the analysis software) seems insufficient to correct for residual tracer activity in successive phantom measurements. In general, subtraction of TACs entails a certain measurement inaccuracy, which seems undesirable for the development of a validation phantom and hampers current MBF computation and phantom reproducibility.

The phantom setup used was not dedicated for measurements with CT; hence, we performed a measurement series upfront for optimization purposes. With minimal adjustments to phantom and flow circuit design, it became possible to perform dynamic perfusion image acquisition using a slightly modified clinical protocol (see Video, Supplemental Digital Content S2). We performed initial perfusion analyses using associated clinical software. Remarkably, the mapped intensity profiles within the mimicked myocardium were higher compared to patient data [28]. This may be due to the made simplifications in phantom design in mimicking the pericardium and myocardium as one. In future research more suitable tissue-mimicking materials will be explored. Possibly this simplified anatomical representation also limits automatic delineation of the heart contours. Fortunately, we could manually delineate VOIs instead, though this type of evaluation is less easy to standardize for future research purposes and therefore less desirable for phantom validation purposes. MBF was subsequently calculated based on the average TICs within these VOIs. These values do not yet correspond to the reference values (Table 2) but do demonstrate the potential of future phantom applications.

Minor adjustments to the phantom setup allowed us to obtain dynamic MR perfusion images according to clinical protocol. The obtained dynamic TIC signals in the LVC and myocardial regions were comparable to those in patients [29]. In this, a simulated perfusion deficit led to a delayed time to peak and a decreased maximum peak intensity compared to surrounding normal perfused tissue (see Figure 7 and Table 2). It should be noted that the perfusion deficit as indicated in segment 2 of Figure 6 and its associated TIC in Figure 7 comprises only half the myocardial phantom region in which the deficit was located. A comprehensive analysis hereof is presented in the software display presented in Supplemental Digital Content S1. These quantitative perfusion measures are still relative and can therefore underdiagnose patients with balanced ischemia or microvascular dysfunction. The utilized MR imaging technique can be further improved, though it is considered sufficient for this proof-of-concept phantom experiment. Within the cardiac MRI field, there are several dynamic imaging methods to look at myocardial perfusion, including calculating MBF. The latter can be performed, for example, with arterial spin labeling [30]. In this technique no contrast agent is used, which greatly reduces the signal-to-noise ratio, but leads to a less robust application. At the moment this application is still in its infancy. Further development of this application can certainly benefit from the dedicated validation phantom as presented in this study.

This study has a few limitations. Only a limited number of measurements were performed per imaging modality (n = 3), which is sufficient for a first feasibility check, though requires more extensive research to draw up an adequate set of requirements for multimodal phantom (re)design. In line with this, for a complete multimodal phantom design it is also important to consider the differences between scanner types and manufacturers as well as clinical perfusion analysis software packages. In addition, better venting capabilities should be incorporated in the phantom design to diminish the presence of air bubbles. For example, small air bubbles were present during phantom measurements in CT, as depicted in the top myocardial region of the image time-lapse in Figure 5. Finally, this phantom feasibility study does not yet include quantitative MPI with contrast enhanced echocardiography. The materials used in current phantom model are not suited for use with ultrasound.

In conclusion, this phantom feasibility study in PET/CT, CT, and MRI has shown that our adapted myocardial perfusion phantom setup offers the potential for multimodal functionality. With the current setup, we were able to successfully perform quantitative perfusion measurements using clinical applications and compare the results to a reference flow measure (indicative, not validated). From these initial phantom measurements, we have gained valuable insights to improve our multimodal phantom design. A next step is to further optimize and validate the mimicking of the respective (radiolabeled) contrast kinetics for each imaging modality.

## Figures and Tables

**Table 1:** Overview of study variables.

Study Variables	PET/CT	CT	MR
*imaging settings*			
scan duration	8.5 min	30 s	60 s
(radiolabeled) contrast medium	400 MBq [^13^N]ammonia	12.6 g iomeprol	7.5 mmol gadolinium
V_tracer_/V_contrast_	10	60	15
v_tracer_/v_contrast_ (mL/s)	1.0	6.0	3.0
V_endflush_ (mL)	30	32	20
v_endflush_ (mL/s)	5.0	6.0	3.0
*phantom settings*			
- myo1	29.0 g zeolite	sponge	7.0 g activated carbon 14.0 g silica granulates
- myo2	14.5 g zeolite 13.7 g silica granulates	sponge	7.0 g activated carbon 14.0 g silica granulates
- myo3	7.3 g zeolite 12.8 g activated carbon	sponge	7.0 g activated carbon 14.0 g silica granulates
Φ_LVC_ (L/min)	4.0	4.0	4.0
Φ_myo1–3_ (mL/min)	[40 80]	[40 80 120]	[60 80]

A_inj_ = injected radiotracer activity; V = volume; v = injection rate; myo1–3 = myocardial tissue regions; LVC = left ventricular cavity; Φ = volume flux.

**Table 2:** Impression of software derived myocardial blood flow (MBF) or other perfusion measures from a reference phantom measurement in PET/CT, CT, and MR.

Imaging Modality	Myocardial Region	Φ (mL/g/min)	MBF (mL/g/min)	TTP (s)
PET/CT	myo1 (HS7)	0.8	0.6	-
	myo2 (HS9)	1.6	0.9	-
	myo3 (HS11)	1.6	0.8	-
CT	myo1 myo2 myo3	1.6 0.8 1.6	4.5 2.8 4.3	- - -
MR	myo1 (s6) myo2 (s4) myo3 (s2)	1.6 1.6 0.8	- - -	32.0 32.0 38.0

TTP = time-to-peak.

## Data Availability

The data presented in this study are available on request from the corresponding author.

## Supplementary Materials

The following supporting information can be downloaded at: https://www.mdpi.com/article/10.3390/bioengineering9090436/s1, Supplemental Digital Content S1: Multimodal perfusion analysis displays using clinical software. Supplemental Digital Content S2: Video that demonstrates contrast distribution in the phantom using dynamic CT myocardial perfusion imaging.
