# Supplementary material for: A Multimodality Myocardial Perfusion Phantom: Initial Quantitative Imaging Results
Source: Bioengineering (Basel). 2022 Sep 4;9(9):436. doi: 10.3390/bioengineering9090436 (PMC9495397; doi:10.3390/bioengineering9090436)
Supplement: Supplementary file 1 [file bioengineering-09-00436-s001.zip › Supplemental Digital Content S1.pdf]

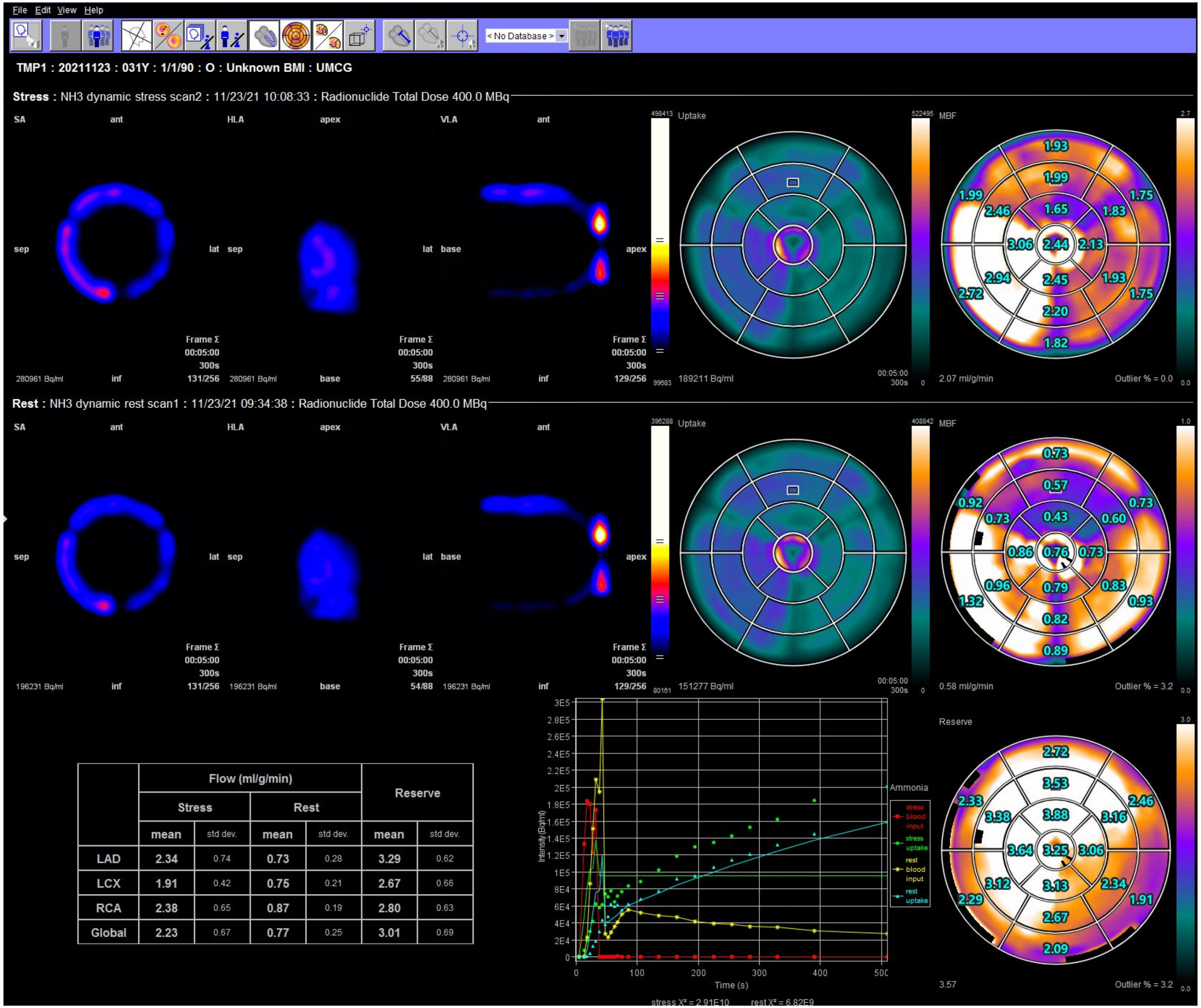

	Flow (ml/g/min)				Reserve	
	Stress		Rest			
	mean	std dev.	mean	std dev.	mean	std dev.
LAD	2.34	0.74	0.73	0.28	3.29	0.62
LCX	1.91	0.42	0.75	0.21	2.67	0.66
RCA	2.38	0.65	0.87	0.19	2.80	0.63
Global	2.23	0.67	0.77	0.25	3.01	0.69

PET/CT perfusion analysis display using SyngoMBF VB14 (Siemens Healthineers, Forchheim, Germany).

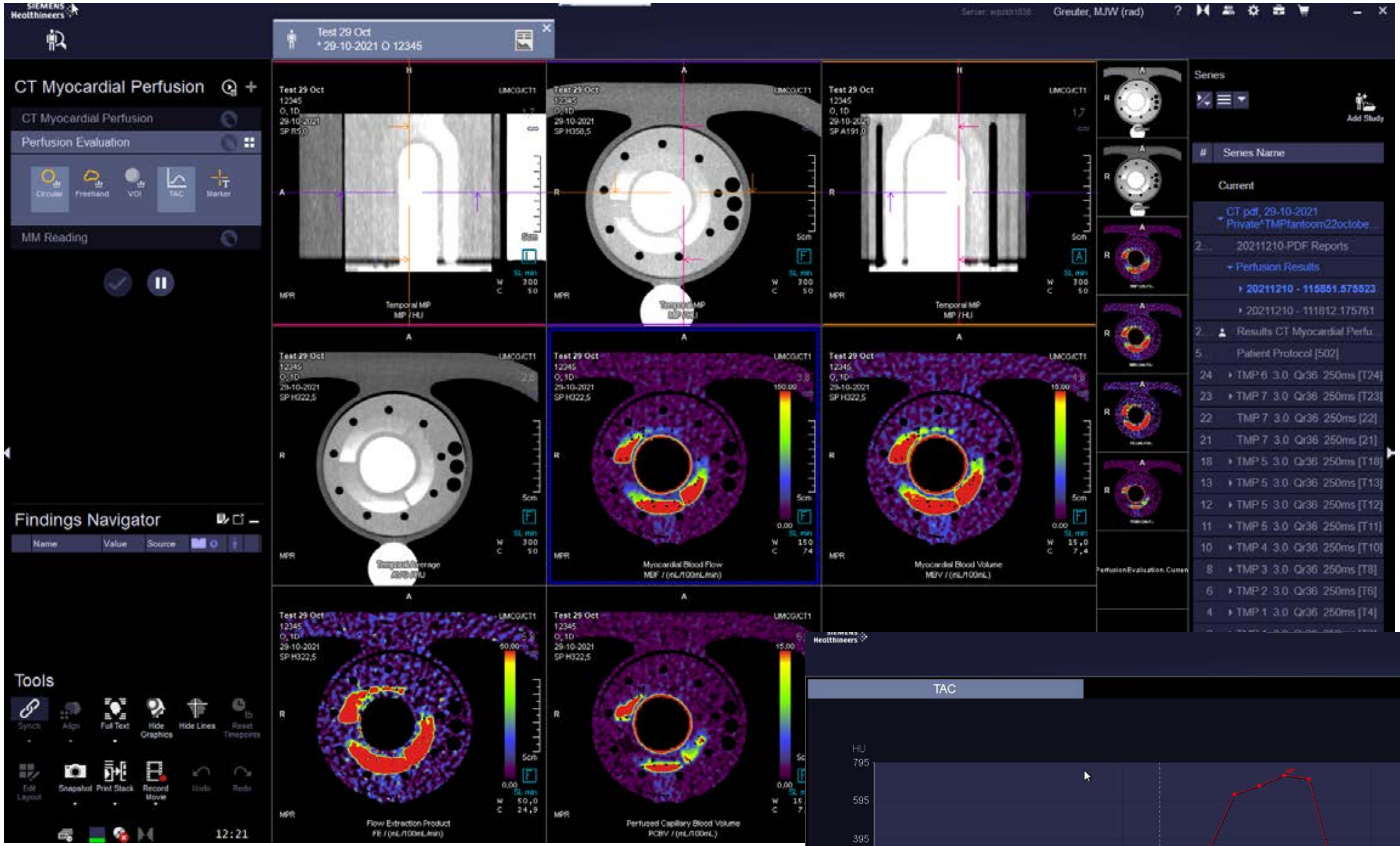

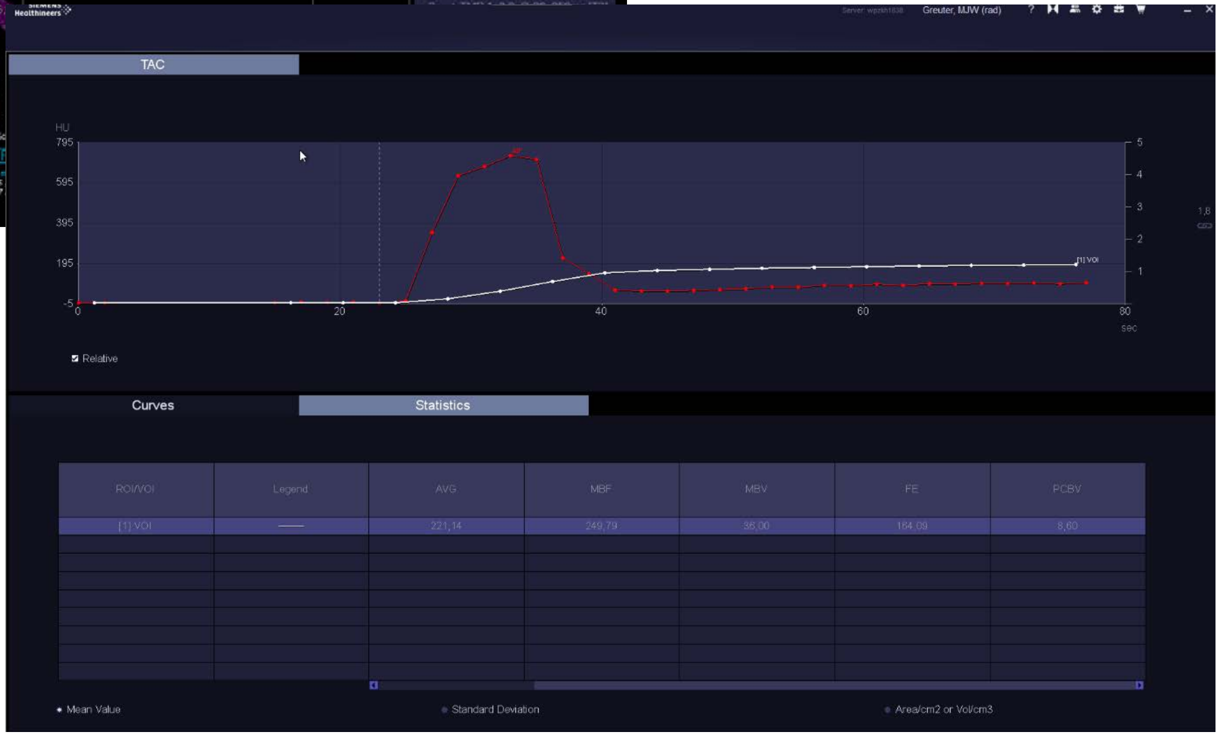

CT perfusion analysis display using Syngo.via Enterprise Browser VB40 HF20 (Siemens Healthineers, Forchheim, Germany).

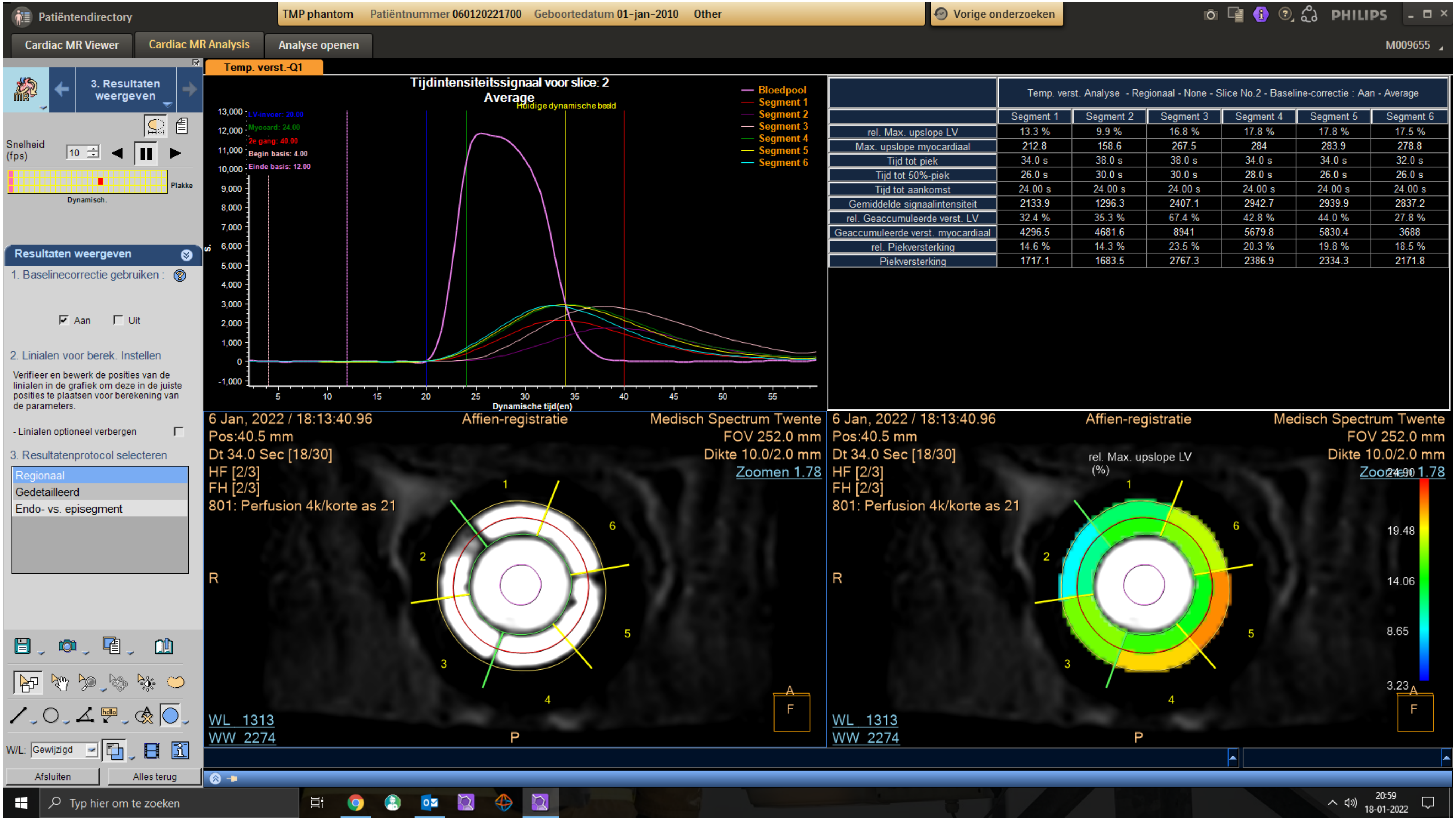

MRI perfusion analysis display using Intellispace software (Philips Healthcare, the Netherlands).
